# Adjustment of sensitisation and challenge protocols restores functional and inflammatory responses to ovalbumin in guinea-pigs

**DOI:** 10.1016/j.vascn.2014.10.007

**Published:** 2015-03

**Authors:** Alexander P.P. Lowe, Kenneth J. Broadley, Anthony T. Nials, William R. Ford, Emma J. Kidd

**Affiliations:** a Cardiff School of Pharmacy, Cardiff University, Redwood Building, King Edward VII Ave, Cardiff CF10 3NB, United Kingdom; b Discovery Biology, Respiratory Centre of Excellence for Drug Discovery, GlaxoSmithKline Medicines Research Centre, Gunnels Wood Road, SG1 2NY Stevenage, United Kingdom

**Keywords:** EAR, early asthmatic response, LAR, late asthmatic response, AHR, airways hyperresponsiveness, Ova, ovalbumin, sGaw, specific airway conductance, Allergic inflammation, Airways hyperresponsiveness, Early asthmatic response, Late asthmatic response, Bronchoconstriction, Ovalbumin

## Abstract

**Introduction:**

Inhalation of antigen in atopic asthma induces early (EAR) and late asthmatic responses (LARs), inflammatory cell infiltration and airways hyperresponsiveness (AHR). Previously, we have established a protocol of sensitisation and subsequent ovalbumin (Ova) inhalation challenge in guinea-pigs which induced these 4 features (Smith & Broadley, 2007). However, the responses of guinea-pigs to Ova challenge have recently declined, producing no LAR or AHR and diminished EAR and cells. By making cumulative modifications to the protocol, we sought to restore these features.

**Methods:**

Guinea-pigs were sensitised with Ova (i.p. 100 or 150 μg) on days 1 and 5 or days 1, 4 and 7 and challenged with nebulised Ova (100 or 300 μg/ml, 1 h) on day 15. Airway function was measured in conscious guinea-pigs by whole-body plethysmography to record specific airway conductance (sGaw). Airway responsiveness to aerosolized histamine (0.3 mM) was determined before and 24 h after Ova challenge. Bronchoalveolar lavage was performed for total and differential inflammatory cell counts. Lung sections were stained for counting of eosinophils.

**Results:**

Lack of AHR and LAR with the original protocol was confirmed. Increasing the Ova challenge concentration from 100 to 300 μg/ml restored AHR and eosinophils and increased the peak of the EAR. Increasing the number of sensitisation injections from 2 to 3 did not alter the responses. Increasing the Ova sensitisation concentration from 100 to 150 μg significantly increased total cells, particularly eosinophils. A LAR was revealed and lymphocytes and eosinophils increased when either the Al(OH)_3_ concentration was increased or the duration between the final sensitisation injection and Ova challenge was extended from 15 to 21 days.

**Discussion:**

This study has shown that declining allergic responses to Ova in guinea-pigs could be restored by increasing the sensitisation and challenge conditions. It has also demonstrated an important dissociation between EAR, LAR, AHR and inflammation.

## 1 Introduction

Asthma is now recognised as a heterogeneous disease with multiple pathologies. Allergic asthma is characterised by early and late asthmatic responses (EARs and LARs) following allergen challenge (O'Byrne, 2009). The EAR is an immediate bronchoconstriction to allergen and usually resolves within the first couple of hours (Leigh et al., 2002). The LAR is a temporally separate and delayed bronchoconstriction, seen in 50% of patients 3–8 h after allergen challenge (Galli, Tsai, & Piliponsky, 2008; O'Byrne, 2009). These responses demonstrate large Inter-subject variability (Kopferschmitt-Kubler, Bigot, & Pauli, 1987), which does not appear to have been examined in animal models. The late asthmatic response is followed by the development of airways hyperresponsiveness (AHR), an increased response to a bronchoconstrictor stimulus such as histamine (Cockcroft & Davis, 2006). These responses are also accompanied by pulmonary inflammation, as manifested by an accumulation of eosinophils, macrophages and lymphocytes in lung parenchyma tissue (Nabe et al., 2005). Specifically, eosinophils are important in the development of late asthmatic responses and AHR (Gauvreau, Watson, & O'Byrne, 1999; Homma, Bates, & Irvin, 2005).

Allergen challenge protocols, using antigens such as ovalbumin (Ova) are used to model characteristics of asthma in guinea-pigs (Buels, Jacoby, & Fryer, 2012; Evans et al., 2012; Lee, Kim, & Kim, 2013). Sensitisation to Ova is usually achieved by intraperitoneal administration with an adjuvant such as aluminium hydroxide (Lindblad, 2004). Animals are then given several weeks for antibodies (immunoglobulins, IgE and IgG) and for immune responses to develop. Re-exposure to Ova, generally by the inhaled route then triggers the effector phase (Chang, Gong, Chen, & Mak, 2011). Lung function can be measured in conscious, spontaneously breathing animals using whole body plethysmography which allows for assessment of multiple functional responses in the same animal over several days.

Mice are the most commonly used species for modelling aspects of asthma, especially inflammation. Guinea-pigs are no longer used as widely but represent valuable models, especially for functional parameters such as the EAR and LAR (reviewed in Canning & Chou, 2008). Guinea-pigs have a similar distribution of mast cells, to humans (Fuchs et al., 2012). Also, the EAR bronchoconstriction is pronounced and mediated by histamine, cysteinyl leukotrienes and prostaglandins in both species, contrasting with mice where the EAR bronchoconstriction is minimal and mediated by 5-HT (Fernandez-Rodriguez, Ford, Broadley, & Kidd, 2008; Moffatt, Cocks, & Page, 2004; Ressmeyer et al., 2006; Zosky et al., 2008). Several groups have demonstrated isolated characteristics of asthma such as AHR, EAR and LAR in guinea-pigs (Riley et al., 2013; Suda et al., 2009). However, most studies do not assess all of these characteristics in the same model together with inflammatory cell recruitment, which has potential limitations for using them to assess drug efficacy of novel treatments (Stevenson & Birrell, 2011). Within this laboratory, a model demonstrating an EAR, LAR, AHR and airway inflammation to Ova challenge in guinea-pigs has been developed (Evans et al., 2012). However, this model has required optimisation on several occasions over the years to continue to produce these features. Lewis, Johnson, and Broadley (1996) modified the allergen challenge conditions to stop the need for mepyramine, which prevents fatal anaphylaxis. Smith and Broadley (2007) modified the sensitisation conditions because of the loss of key features over time. They increased the amount of Ova used and the number of injections given. This restored the EAR, LAR and AHR to Ova challenge. Five years later, at the beginning of the present study the responses had again waned with a loss of the LAR and AHR.

The aim of this study was to re-establish an acute guinea-pig model of asthma displaying early and late asthmatic responses, airway hyperresponsiveness and airway inflammation as demonstrated by Smith and Broadley (2007) and Evans et al. (2012).

## 2 Materials and methods

All chemicals were obtained from Sigma-Aldrich, UK or Fisher-Scientific, UK unless stated otherwise.

### 2.1 Animal husbandry

Male Dunkin-Hartley guinea-pigs, 200–300 g were purchased from Harlan Ltd, UK or Charles River, Germany. Guinea-pigs were housed in pathogen free conditions with 12 h light/dark cycles. All procedures were carried out in accordance with Home office license conditions of the Animals (Scientific Procedures) Act 1986 covering animal husbandry and severity limits and EU Directive 2010/63/EU for animal experiments.

### 2.2 Ovalbumin sensitisation and challenge

Guinea-pigs were sensitised by bilateral intra-peritoneal injections of a solution containing ovalbumin (Ova), VWR (catalogue number 20771.236, UK, 100 or 150 μg) and aluminium hydroxide (Al(OH)_3_, Sigma-Aldrich, UK, 100 or 150 mg) in 1 ml of normal saline on days 1 and 5 or days 1, 4 and 7.

Guinea-pigs were exposed to inhaled ovalbumin (100 μg/ml or 300 μg/ml) on days 15 or 21. Exposure was performed in a Perspex exposure chamber (15 × 30 × 15 cm) using a DeVilbiss nebuliser, delivered at a rate of 0.3 ml/min-1 and at an air pressure of 20 ib p.s.i. Guinea-pigs were exposed for 1 h. Control groups of guinea-pigs were sensitised by the same protocols and exposed to aerosolised saline. Lung function was recorded at intervals for 12 h and at 24 h post-challenge, the animals being removed from the chamber after each determination.

### 2.3 Ovalbumin protocols

Six different Ova sensitisation and challenge conditions were used based on the original protocol of Smith and Broadley (2007). This protocol is referred to as protocol 1. Changes were made cumulatively from protocols 1 to 5. Protocol 6 is a modification of protocol 4 (Table 1).

### 2.4 Non-invasive measurement of specific airway conductance

Airway function was measured in conscious, spontaneously breathing guinea-pigs using non-invasive double chamber plethysmography (PY-5551, Buxco systems, USA) to measure specific airway conductance (sGaw).

### 2.5 Airway response to histamine

Airway responses to aerosolized histamine were determined before and 24 h after Ova challenge using whole body plethysmography. Histamine (0.3 mM) was nebulised (Buxco nebuliser) direct to the nasal component of the plethysmograph chamber at a rate of 0.5 l per minute, 2 min nebulisation, and 10% duty setting per chamber. This nebulizer protocol evokes minimal bronchoconstriction in naïve guinea-pigs and before Ova challenge of sensitised animals. Lung function was measured before histamine inhalation and at 0, 5 and 10 min post-histamine exposure.

### 2.6 Pulmonary inflammation

Following the final histamine challenge, guinea-pigs were sacrificed by an intra-peritoneal overdose of sodium pentobarbitone (Euthatal 400 mg/kg). Guinea-pigs were then bled via severance of a carotid artery and subsequently a polypropylene cannula was inserted into the trachea. Bronchoalveolar lavage was performed using normal saline (1 ml per 100 g of guinea-pig weight) instilled through the cannula for 3 min before withdrawal. This process was then repeated, the samples pooled and total number of cells/ml counted using a Neubauer haemocytometer. Differential cell counts were performed after centrifuging 100 μl of undiluted lavage fluid using a Shandon cytospin onto glass microscope slides, at 110 g for 7 min. Slides were subsequently stained with 1.5% Leishman's solution in 100% methanol for 6 min. Leukocyte subpopulations counted included eosinophils, macrophages, lymphocytes and neutrophils. A minimum of 200 cells per slide were counted.

### 2.7 Tissue eosinophilia

Lung lobe samples were stored in 4% formaldehyde and 1–2 mm bilateral sections cut. Samples were dehydrated in increasing concentrations of ethanol and then chloroform. Tissue sections were then set into wax blocks using molten paraffin. 5 μm sections were cut using a microtome and mounted on poly-L-lysine-coated slides. Slides were stained using the Sirius red staining protocol which allows the identification of eosinophils (Meyerholz, Griffin, Castilow, & Varga, 2009). The number of eosinophils was counted per field of view magnification. Four fields of view were counted per animal. Eosinophils were defined as cells demonstrating a cytoplasm staining an intense red with dark bi-lobed nuclei.

### 2.8 Data analysis

All lung function data were plotted as a percentage of baseline to take into account the individual differences in guinea-pig baseline sGaw values. To account for differences in the timing of allergen responses during the early (0–6 h) and late (6–12 h) phases, sGaw was also expressed as the peak bronchoconstriction, displayed as a histogram next to a time course plot. Results are plotted as the mean ± standard error of the mean (SEM). Student's t-tests were used for the comparison of differences between groups or data points. One way analysis of variance (ANOVA) followed by a Dunnett's post-test was used when 2 or more groups were being compared to a control group. A p value less than 0.05 was considered significant.

## 3 Results

### 3.1 Effect of Ova sensitisation and challenge on airway function

**Fig. 1:**
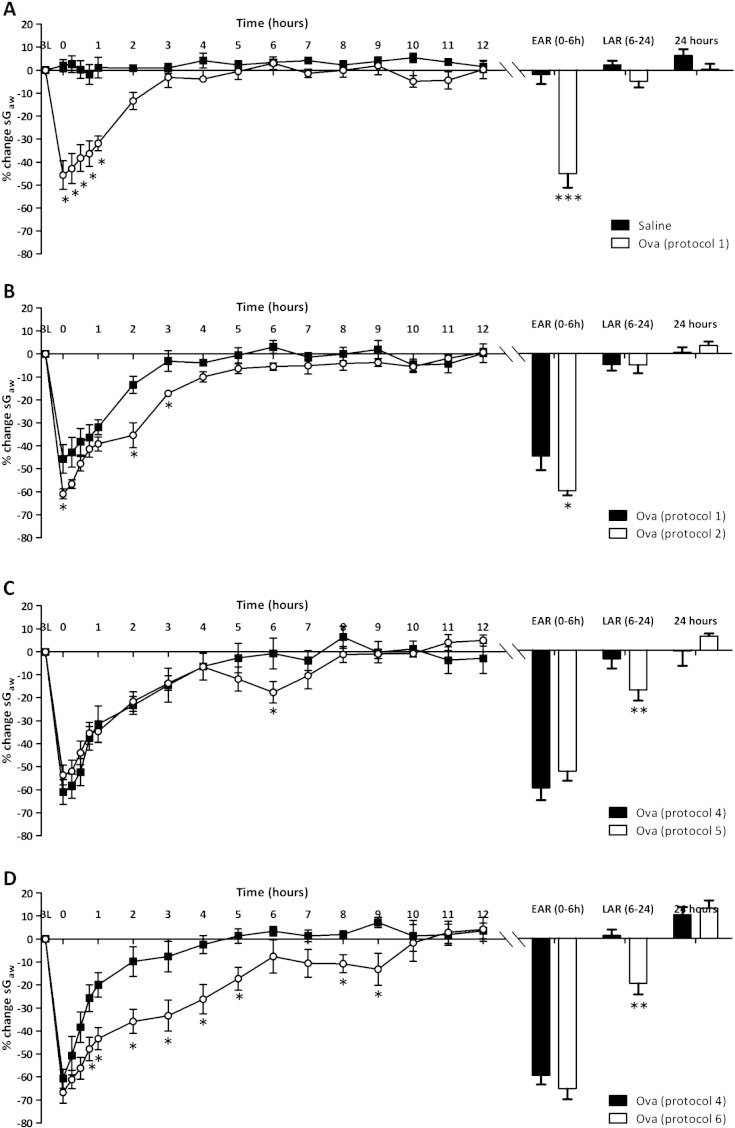
The mean time-courses for sGaw values in guinea-pigs sensitised with 2 injections of 100 μg/ml ovalbumin (Ova) and 100 mg Al(OH)_3_ and challenged on day 15 with saline (saline), 100 μg/ml (protocol 1), 300 μg/ml Ova (protocol 2), sensitised with 3 injections of 150 μg ovalbumin (Ova) and 100 mg Al(OH)_3_ and challenged on day 15 with 300 μg/ml Ova (protocol 4), 3 injections of 150 μg ovalbumin (Ova) and 150 mg Al(OH)_3_ and challenged on day 15 with 300 μg/ml (protocol 5) or 3 injections of 150 μg ovalbumin (Ova) and 100 mg Al(OH)_3_ on day 21 (protocol 6). The histogram represents the maximum bronchoconstriction values recorded during the early asthmatic response (EAR) (0 –6 h) and late asthmatic response (LAR) (6–12 h). Mean changes in sGaw are expressed as mean ± SEM percentage change from baseline. A negative value represents a bronchoconstriction. N = 6; * significantly different from paired protocol p < 0.05, **p < 0.01, ***p < 0.001 performed with a two tailed Student's t-test.

Fig. 1 represents the mean time-course changes in sGaw over 24 h following Ova challenge in conscious guinea-pigs sensitised and challenged with saline or protocols 1–6.

The sensitisation and challenge protocol previously used successfully in this laboratory (Evans et al., 2012; Smith & Broadley, 2007) was protocol 1, which consisted of sensitisation with 2 injections of 100 μg/ml Ova and 100 mg Al(OH)_3_, with subsequent 100 μg/ml Ova challenge. This resulted in an immediate significant reduction in sGaw (− 45.6 ± 6.2%), characteristic of an early asthmatic response (Fig. 1A). This bronchoconstriction did not return to saline-challenged levels until 2 h post-challenge. No further decreases in sGaw, characteristic of the late asthmatic response, were observed. Increasing the Ova challenge concentration to 300 μg/ml (protocol 2, Fig. 1B) increased the immediate bronchoconstriction (− 60.9 ± 2.1%), compared to protocol 1, which returned to baseline levels 4 h post-challenge. No late asthmatic response was observed. Increases in the Ova sensitisation concentration to 150 μg/ml (protocol 4) and the number of injections (protocol 3) did not alter the airway response (not shown).

Increasing the Al(OH)_3_ adjuvant concentration to 150 mg (protocol 5, Fig. 1C) did not alter the size or duration of the early asthmatic response compared to protocol 4 but produced a late asthmatic response, characterised by a significant decrease in sGaw at 6 h (− 17.6 ± 4.6% compared to − 3.8 ± 4.2%). Increasing the time between Ova sensitisation and challenge, while returning to protocol 4 conditions (protocol 6, Fig. 1D) significantly increased the duration of the early asthmatic response compared to protocol 4, with a significant decrease in sGaw at all time points from 45 min to 5 h post-challenge. This group also demonstrated a late asthmatic response between 8 and 9 h. The mean peak response during this period was − 19.9 ± 4.9% compared to protocol 4, 1.3 ± 2.6%.

### 3.2 Effect of Ova sensitisation and challenge on airway response to histamine

**Fig. 2:**
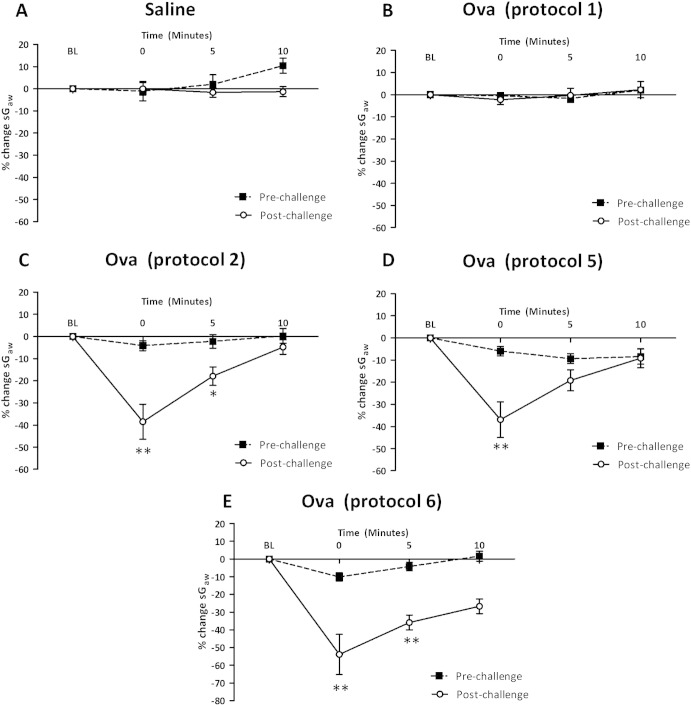
Response of the airways to nebulised histamine delivered in a plethysmograph (0.3 mM, 10% duty cycles with 0.5 LPM flow per chamber over 2 min, 1 min drying period) to guinea-pigs sensitised with 2 injections of 100 μg/ml ovalbumin (Ova) and 100 mg Al(OH)_3_ and challenged with on day 15 with saline (saline), 100 μg/ml Ova (protocol 1), 300 μg/ml Ova (protocol 2), 3 injections of 150 μg ovalbumin (Ova) and 150 mg Al(OH)_3_ and challenged on day 15 with 300 μg/ml (protocol 5) or sensitised with 3 injections of 150 μg ovalbumin (Ova) and 100 mg Al(OH)_3_ on day 21 (protocol 6). Values were recorded 24 pre- and post-Ova or saline challenge. Mean changes in sGaw are expressed as mean ± SEM percentage change from baseline. A negative value represents a bronchoconstriction. N = 6; * significantly different from time-paired pre-Ova challenge values p < 0.05, **p < 0.01; performed with a two tailed Student's t-test.

No significant bronchoconstriction to histamine was observed in any experimental animal 24 h before Ova or saline challenge (Fig. 2). Small changes were observed in some groups which represent the normal variation in sensitivity to a threshold concentration of histamine. In animals challenged with saline, no histamine-induced bronchoconstriction was observed 24 h after saline (Fig. 2A). Animals sensitised with 2 injections of 100 μg/ml Ova and 100 mg Al(OH)_3_ and challenged with 100 μg/ml Ova (protocol 1, Fig. 2B) also lacked histamine-induced bronchoconstriction, indicating the absence of AHR. Increasing the Ova challenge concentration to 300 μg/ml (protocol 2, Fig. 2C) caused a significant bronchoconstriction to histamine 24 h after Ova challenge (− 38.5 ± 7.9% compared to pre- − 4.1 ± 2.3%) which resolved within 10 min. Increasing the Al(OH)_3_ concentration (protocol 5, Fig. 2D), increasing Ova sensitisation concentration (protocol 4) and the number of injections (protocol 3) did not further alter the nature of this response (data not shown).

Increasing the time between Ova sensitisation and challenge (protocol 6, Fig. 2E) increased the size of the immediate bronchoconstriction to histamine 24 h post-challenge (− 53.9.4 ± 11.4%) compared to pre-Ova challenge, (− 10.1 ± 2.4%). The duration of the bronchoconstriction was also increased, at 10 min into the response, the bronchoconstriction was − 26.7 ± 11.4% compared to the pre-Ova challenge level of 1.6 ± 2.7%.

### 3.3 Effect of Ova sensitisation and challenge on pulmonary inflammation

**Fig. 3:**
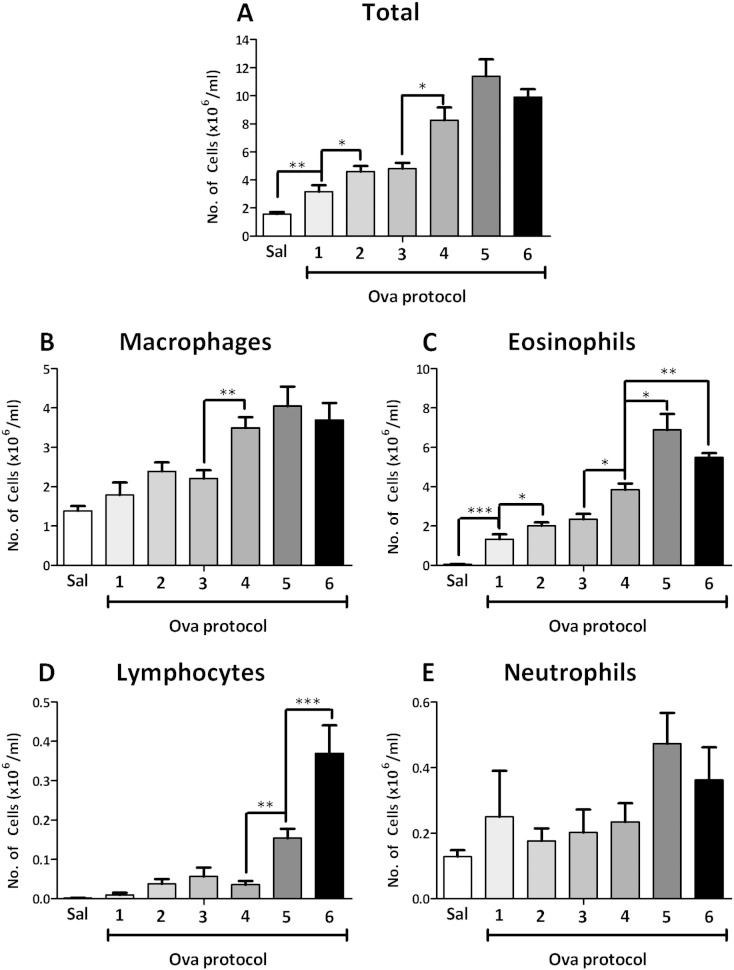
The total cell (A), macrophage (B), eosinophil (C), lymphocyte (D) and neutrophil (E) counts in bronchoalveolar fluid in guinea-pigs sensitised with 2 injections of 100 μg/ml ovalbumin (Ova) and 100 mg Al(OH)_3_ and challenged with on day 15 with saline (sal), 100 μg/ml (protocol 1), 300 μg/ml Ova (protocol 2), sensitised with 3 injections of 100 μg/ml ovalbumin (Ova) and 100 mg Al(OH)_3_ and challenged on day 15 with 300 μg/ml Ova (protocol 3), sensitised with 3 injections of 150 μg ovalbumin (Ova) and 100 mg Al(OH)_3_ and challenged on day 15 with 300 μg/ml Ova (protocol 4), 3 injections of 150 μg ovalbumin (Ova) and 150 mg Al(OH)_3_ and challenged on day 15 with 300 μg/ml (protocol 5) or 3 injections of 150 μg ovalbumin (Ova) and 100 mg Al(OH)_3_ on day 21 (protocol 6). N = 6; * significantly different from the previous protocol (protocol 4 in the case of protocol 6) p < 0.05, **p < 0.01, ***p < 0.001; performed with a two tailed Student's t-test.

100 μg/ml Ova challenge significantly increased total lavage cells (protocol 1, Fig. 3A, 3.2 ± 0.5 × 106/ml) compared to saline (1.6 ± 0.13 × 106/ml). Eosinophils (Fig. 3C) made up most of this increase (1.3 ± 0.3 × 106/ml) compared to saline (0.05 ± 0.01 × 106/ml). Increasing the Ova challenge concentration (protocol 2) significantly increased the total cell numbers (5.3 ± 0.4 × 106/ml) compared to protocol 1 (3.2 ± 0.5 × 106/ml). Eosinophils were significantly elevated (2.0 ± 0.2 × 106/ml) compared to protocol 1 (1.3 ± 0.3 × 106/ml). Increasing the number of 100 μg Ova sensitisation injections (protocol 3) had no effect on any cell type measured.

Increasing the Ova sensitisation concentration to 150 μg (protocol 4) significantly increased total cells (8.3 ± 0.9 × 106/ml) compared to protocol 3 (4.8 ± 0.4 × 106/ml). Eosinophils (3.9 ± 0.3 × 106/ml compared to 2.4 ± 0.3 × 106/ml) and macrophages (Fig. 3B, 3.5 ± 0.3 × 106/ml compared to 2.2 ± 0.2 × 106/ml) were also significantly increased. Increasing the Al(OH)_3_ sensitisation concentration to 150 mg (protocol 5) significantly increased eosinophils (6.9 ± 0.8 × 106/ml) compared to protocol 4 (4.6 ± 0.5 × 106/ml). Lymphocytes (Fig. 3D) were also significantly increased (0.15 ± 0.02 × 106/ml) compared to protocol 4 (0.3 ± 0.01 × 106/ml).

Increasing the duration between Ova sensitisation and challenge (protocol 6) to 21 days did not significantly change the total cell numbers. Lymphocytes (0.37 ± 0.07 × 106/ml) and eosinophils (5.5 ± 0.2 × 106/ml) were significantly increased compared to animals challenged on day 15 (protocol 4, 0.04 ± 0.01 × 106 and 3.9 ± 0.3 × 106/ml, respectively). Neutrophils (Fig. 3E) were unchanged in all protocols.

### 3.4 Effect of Ova sensitisation and challenge on tissue eosinophilia

**Fig. 4:**
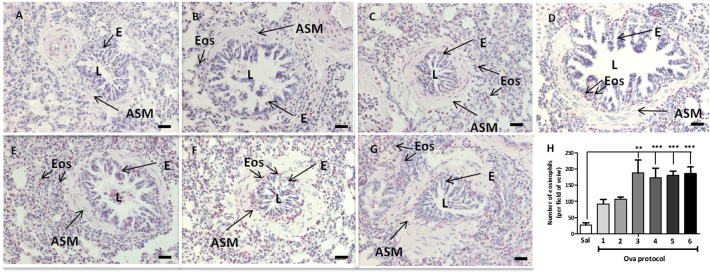
Guinea-pig lung tissue sections stained with Sirius red. Sensitisation and challenge varied accordingly A 2 × 100 μg/ml Ova/100 mg Al(OH)_3_ with saline challenge (saline). B 2 × 100 μg/ml Ova/100 mg Al(OH)_3_ with 100 μg/ml Ova challenge (protocol 1). C 2 × 100 μg/ml Ova/100 mg Al(OH)_3_ with 300 μg/ml Ova challenge (protocol 2). D 3 × 100 μg/ml Ova/100 mg Al(OH)_3_ with 300 μg/ml Ova challenge (protocol 3). E 3 × 150 μg Ova/100 mg Al(OH)_3_ with 300 μg/ml Ova challenge (protocol 4). F 3 × 150 μg Ova/150 mg Al(OH)_3_ with 300 μg/ml Ova challenge (protocol 5). G 3 × 100 μg/ml Ova/100 mg Al(OH)_3_ with 300 μg/ml Ova challenge on day 21 (protocol 6). H the number of tissue eosinophils (per field of view). Original magnification 200×; bar = 50 μm). L: lumen; ASM: airway smooth muscle; E: epithelium. Eosinophils (Eos) were defined as cells demonstrating a cytoplasm staining an intense red with dark bi-lobed nuclei. N = 4–6; ** significantly different to saline p < 0.01; ***p < 0.001; performed with one-way analysis of variance with Dunnett's post-test.

Fig. 4A–G shows typical photomicrographs for lung sections stained with Sirius red to identify eosinophils. Fig. 4H shows the number of eosinophils counted per field of view. A progressive trend for increased eosinophil numbers was observed with cumulative modifications to the Ova sensitisation and challenge protocol. This reached significance compared to saline when the number of sensitisation injections was increased to 3 (187.4 ± 40.2, saline: 27.0 ± 7.4). All subsequent modifications maintained elevated eosinophilia compared to saline but did not further increase it (173.7 ± 29.1, 180.2 ± 13.0 and 185.8 ± 20.5 respectively).

### 3.5 Variability of airway responses to Ova

**Fig. 5:**
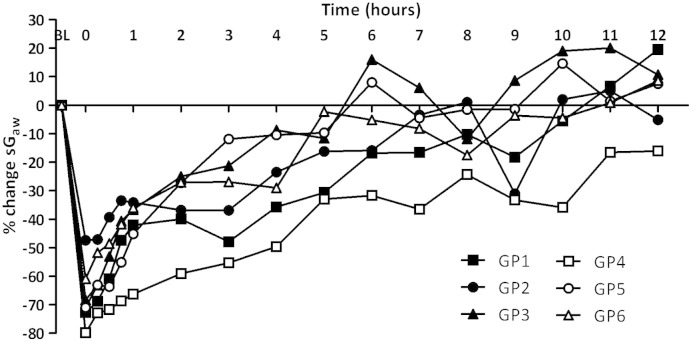
The time-course values of sGaw in individual guinea-pigs sensitised with 3 injections of a suspension of 150 μg Ova and 100 mg Al(OH)_3_ and challenged with 300 μg/ml Ova on day 21 (protocol 6). Changes in sGaw are expressed as percentage change from baseline. A negative value represents a bronchoconstriction. The figure illustrates the individual variability in the early and late asthmatic responses to Ova challenge in guinea-pigs.

Fig. 5 demonstrates the variability between guinea-pigs in the timing of the early and late asthmatic responses, exemplified by data from the final sensitisation and challenge protocol used (protocol 6). Each guinea-pig displays a different EAR and LAR temporal profile.

## 4 Discussion

This study has confirmed the loss over time of essential features of asthma in a guinea-pig model that had previously shown early and late asthmatic responses, AHR and airway inflammation. By making cumulative modifications to the allergen sensitisation and challenge conditions, however, it has been possible to restore these four features of the model.

Sensitisation of guinea-pigs with 2 injections of 100 μg/ml Ova and 100 mg Al(OH)_3_ and subsequent Ova challenge on day 15 with 100 μg/ml Ova (protocol 1) did not evoke a LAR or AHR. A small early phase immediately after allergen challenge and increased eosinophil influx compared to saline challenge were observed. This protocol had previously been effective at producing the full range of allergic responses (Evans et al., 2012; Smith & Broadley, 2007). The present work suggests that there has been a progressive loss of sensitivity of guinea-pigs to ovalbumin over time. The reason for the deterioration of allergic responses remains unknown although it does not appear to be related to any changes in diet, shipping, ovalbumin or season. The process does seem to be an ongoing phenomenon as we have reported the need for modifications on two previous occasions (Lewis et al., 1996; Smith & Broadley, 2007).

Increasing the Ova challenge concentration 3-fold increased the peak bronchoconstriction of the EAR and induced AHR 24 h after allergen challenge. A further increase in total cell and eosinophil numbers was seen. The addition of an extra sensitisation injection did not alter functional responses but increased bronchial tissue eosinophil numbers, with no significant change in lavage eosinophil numbers. Similarly, increasing the Ova sensitisation concentration did not alter functional responses but did increase total and eosinophil lavage numbers.

Having increased the Ova sensitisation and challenge concentrations, either increasing the Al(OH)_3_ concentration during sensitisation or increasing the duration between Ova sensitisation and challenge was able to induce the full range of functional and inflammatory responses; EAR, LAR, AHR and pulmonary inflammation. The increase in Al(OH)_3_ concentration revealed a LAR at 6 h post-allergen challenge, lasting for 1 h. Extending the time between allergen sensitisation and challenge prolonged the EAR and LAR, the latter characterised by a bronchoconstriction lasting 2 h. AHR to histamine was more pronounced in guinea-pigs with an increased duration between sensitisation and challenge but not significantly so. This protocol also significantly increased lymphocyte numbers when compared to increasing the Al(OH)_3_ concentration.

Therefore, 3 injections of 150 μg Ova and 100 mg Al(OH)_3_ followed by 300 μg/ml Ova challenge on day 21 can be seen to produce an EAR and LAR, a robust AHR to histamine and elevated macrophage, lymphocyte and eosinophil numbers in lavage and eosinophils in the bronchi.

The early asthmatic response was consistently observed with all protocols and therefore appears to be reliably induced by lower levels of sensitisation and challenge. Allergen challenge in sensitised animals causes mast cell degranulation by the crosslinking of FcεR1 receptors, releasing histamine, leukotrienes, prostaglandins and platelet activating factor which mediate the EAR bronchoconstriction (Beasley et al., 1989; Björck & Dahlén, 1993; Smith, Rubin, & Patterson, 1988; Zielen et al., 2013). We believe that the immediate fall in sGaw seen with this model represents the EAR since earlier studies with this model show that it is associated with histamine release (Toward & Broadley, 2004). Furthermore, the EAR is resistant to corticosteroids which reduce the LAR (Evans et al., 2012). In the present study, increasing the Ova challenge dose 3-fold increased the magnitude of the immediate bronchoconstriction, possibly as a result of increased FcεR1 crosslinking and release of bronchoconstrictor substances (Frandsen, Krohn, Hoffmann, & Schiøtz, 2013; MacGlashan, 1993). Smith and Broadley (2007) demonstrated that increasing the concentration of Ova used in sensitisation can also further decrease sGaw immediately after allergen challenge. This was possibly due to enhanced IgE production following sensitisation (Frandsen et al., 2013).

Mast cells and basophils release a range of additional factors including cytokines, chemokines and growth factors during the EAR, which have a role in later events such as lymphocyte activation and eosinophil influx (Amin, 2012; Bradding et al., 1994; Nouri-Aria et al., 2001). In turn, inflammatory cells release further mediators including TNF-α and eosinophil cationic protein which increase sensitivity to bronchoconstrictor agents (Homma et al., 2005; Makwana, Gozzard, Spina, & Page, 2012). The latter, released from eosinophils, can damage the epithelium and expose underlying sensory nerves, increasing sensitivity to bronchoconstrictor stimuli like histamine (Homma et al., 2005). In the present study, lavage eosinophil numbers increased at 24 h, concomitant with the development of AHR. However, this relationship is not clear cut since the original Ova protocol used in this study (protocol 1) resulted in significant eosinophilia but with no AHR. Similarly, in other models and humans, eosinophilia and AHR have been observed to be dissociated (Birrell, Battram, Woodman, McCluskie, & Belvisi, 2003; Leckie et al., 2000). Cell counts can differ between lavage fluid and lung sections which could explain this result (Maestrelli et al., 1995). However, it was observed in this study that eosinophil numbers were moderately related between assessment methods, although tissue assessment seemed less likely to discern small changes. This suggests that the number of eosinophils may not be important in AHR. It does not discount that some other factor such as eosinophil activation status could be more critical. The AHR observed in the present study can be assumed to be non-specific as previous studies with earlier version of our model have shown increases in sensitivity to a wide range of spasmogens (Spruntulis & Broadley, 1999).

Allergen sensitisation begins with the uptake of antigen by antigen presenting cells (APCs) which process and present it to lymphocytes, which in turn undergo either apoptosis or activation (Hammad et al., 2010). Activation leads to the development of an allergic immune response. The extent of this response is dependent on the sensitisation conditions. Increased immune stimulation during sensitisation results in increased lymphocyte priming and consequently stronger responses when the allergen is re-encountered. In the present study, cumulative modifications to the sensitisation conditions including increased number of injections, Ova concentration and Al(OH)_3_ concentration caused a progressive increase in total and eosinophil counts. Al(OH)_3_ enhances sensitisation to antigens via a variety of mechanisms including enhanced antigen uptake, T-cell proliferation, uric acid formation, inflammasome formation and promotion of Th2 type responses (Eisenbarth et al., 2002; Kool et al., 2008; Morefield et al., 2005; Sokolovska, Hem, & HogenEsch, 2007). In accordance with this, increased Al(OH)_3_ concentration significantly increased lymphocyte influx and induced the development of a LAR, suggestive of enhanced sensitisation. Al(OH)_3_ produces these effects in a concentration-dependent manner, with an excess of free adjuvant required for increased immune stimulation (Majgaard Jensen & Koch, 1988).

Allergen sensitisation takes several weeks to develop, involving the production of IgE and activation of lymphocytes. Increasing the time between allergen sensitisation and challenge in the current study increased several of the parameters measured including the duration of the EAR, lymphocyte and eosinophil numbers and induced a LAR. This suggests that in previous protocols allergen sensitisation was still ongoing during challenge and an increased period between the two was required for the generation of a full response. This modification restores the gap between sensitisation and challenge to the duration used in this laboratory's original sensitisation protocol (Lewis et al., 1996) which had decreased with previous modifications (Smith & Broadley, 2007). Notwithstanding the reduced time between final sensitisation dose and challenge when increasing to 3 sensitisations, there was still a loss of allergic responses with protocol 1 compared to previous studies. The addition of a 3rd sensitisation injection on day 7 resulted in a further shortening of the sensitisation period to 8 days. 8 days between the final allergen sensitisation and challenge may not be enough time to produce a full immunological response, except when the sensitisation conditions are increased to a certain extent, as seen in guinea-pigs sensitised with an increased adjuvant concentration.

The late asthmatic response is associated with an influx of a range of inflammatory cells including eosinophils and T lymphocytes (Nabe et al., 2005). Eosinophilia is correlated with the magnitude of the LAR, both being significantly increased in humans and animal models following allergen challenge (Dente et al., 2008; Evans et al., 2012; Gauvreau et al., 1999). Additionally, corticosteroids which reduce eosinophil and lymphocyte numbers also decrease the LAR (Kawayama et al., 2008; Leigh et al., 2002). The present study demonstrated that increases in both eosinophils and lymphocytes coincided with the development of a LAR, supporting a link between these parameters. Although we examined cellular influx at 24 h after Ova challenge and not at the peak of the LAR, our previous studies with earlier versions of this model have shown significant increases in neutrophils, macrophages and eosinophils at the time of the LAR (Danahay, Broadley, McCabe, Nials, & Sanjar, 1999; Toward & Broadley, 2004). However, not all results from this study support this relationship; eosinophils were also increased in protocols 1–4, which did not demonstrate a LAR. Studies in humans have also demonstrated similar results. Blocking OX40, a co-stimulator receptor important in generating allergic responses significantly attenuated eosinophilia with no effect on the LAR (Gauvreau et al., 2014). Additionally, older studies have demonstrated that anti-IL-5 therapy reduced eosinophilia but not AHR and the LAR in humans (Leckie et al., 2000). Overall, the role of eosinophils in the LAR remains uncertain. The investigation of factors such as the activation status of eosinophils may be more revealing than cell number. Substances released from eosinophils including platelet activating factor and eosinophil cationic protein can directly cause LAR bronchoconstriction and therefore may correlate better with LAR (Evans, Barnes, Cluzel, & O'Connor, 1997; Gauvreau et al., 2011). There are also recent suggestions that central reflexes may drive a LAR in some models of allergen challenge in guinea-pigs (Smit et al., 2014).

Functional responses to allergens demonstrate low intra-subject but high inter-subject variation in humans (Kopferschmitt-Kubler et al., 1987). The reasons for this variability are likely to be multifactorial including gender and total and allergen-specific IgE levels (Petersen, Mosbech, & Skov, 1996). Examination of the individual guinea-pig responses in the final protocol of the present study highlights how this phenomenon is also observed in animal models. This emphasises the need for including sufficient numbers in experimental groups to have sufficient statistical power, as well as multiple measurements to evaluate peak responses over a wide temporal window.

In conclusion, this study has demonstrated a dissociation between eosinophil influx and LAR as well as AHR. It has highlighted that assessing parameters in isolation, such as inflammatory cell influx in bronchoalveolar lavage fluid, would fail to identify if other key components of the allergic response and its functional outcomes (e.g. AHR) are absent. These models would be inadequate for examining the complex relationship between inflammatory and functional parameters that would be required in preclinical testing of novel therapeutics or identification of potential therapeutic mechanisms. Finally, we achieved our objective of restoring a full profile of functional and inflammatory responses by manipulating the sensitisation and challenge protocols.

## Author contributions

An equal contribution to the original idea, study design, analysis and preparation by Alexander Lowe, Anthony Nials, William Ford, Emma Kidd and Kenneth Broadley. The experimental contribution was made by Alexander Lowe.

## Figures and Tables

**Table 1:**
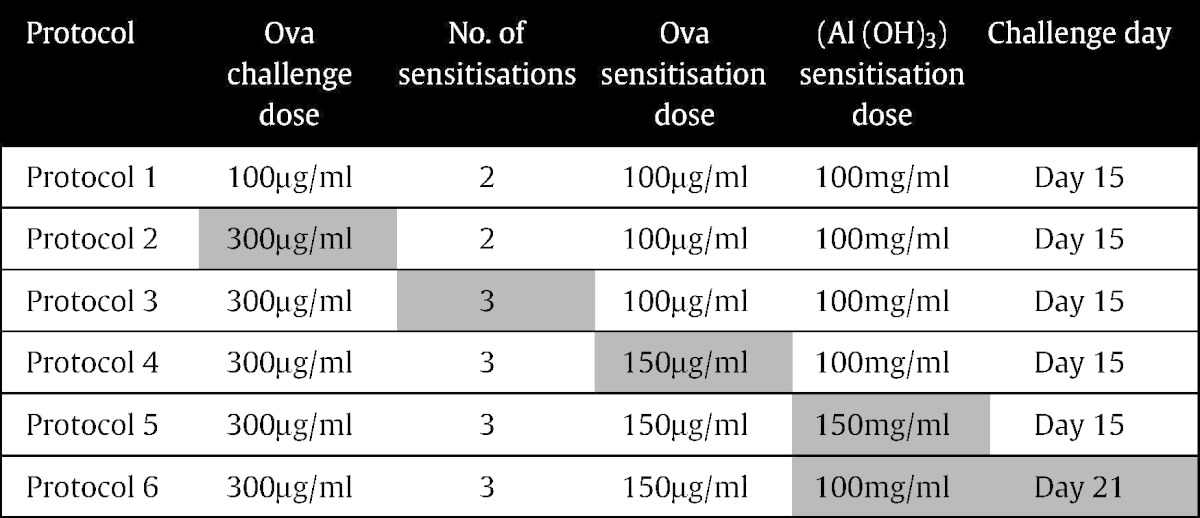
The six ovalbumin protocols used, with cumulative modifications from the previous protocol highlighted in grey shading. Protocol 6 is a modification of protocol 4.
